# Overexpression of *Malus baccata WRKY63* Enhances Cold Tolerance by Increasing the Antioxidant Level Associated with ROS Scavenging

**DOI:** 10.3390/ijms262411997

**Published:** 2025-12-12

**Authors:** Wanda Liu, Tianhe Wang, Xinhui Wang, Zhiwei Wang, Baitao Guo, Yu Wang, Xiaoyu Shen, Jilong Han, Wenhui Li, Deguo Han

**Affiliations:** 1 Key Laboratory of Biology and Genetic Improvement of Horticultural Crops (Northeast Region)/National-Local Joint Engineering Research Center for Development and Utilization of Small Fruits in Cold Regions, Ministry of Agriculture and Rural Affairs, College of Horticulture & Landscape Architecture, Northeast Agricultural University, Harbin 150030, China; liuwanda@haas.cn (W.L.); wxh18846915926@163.com (X.W.); 2 Horticulture Branch, Heilongjiang Academy of Agricultural Sciences, Harbin 150040, China; haaswth@126.com (T.W.); lyjg111@163.com (Z.W.); 18904650420@163.com (B.G.); haaslwd@126.com (Y.W.); 13029921528@163.com (X.S.); hanjilong0000@163.com (J.H.)

**Keywords:** *Malus baccata* (L.) Borkh, *MbWRKY63*, low-temperature stress, genic transformation, transcriptional control

## Abstract

During their natural growth, plants encounter adverse environmental conditions, such as chilling injury, freezing injury, drought, and salt damage, collectively known as abiotic stresses. Several studies have shown that WRKY proteins regulate various abiotic stress responses and plant developmental processes. However, researchers have rarely investigated WRKY genes associated with the stress response in apples. Within this research, *Malus baccata* (L.) Borkh as the experimental material. We isolated and cloned *MbWRKY63* and investigated its function in low-temperature stress tolerance. Subcellular localization analysis shows that MbWRKY63 localizes to the cell nucleus. Tissue-specific expression analysis revealed that *MbWRKY63* is relatively highly expressed in the young leaves and root tissues of apples. Under low-temperature treatment at 4 °C, *Arabidopsis thaliana* plants that overexpressed *MbWRKY63* showed greater cold stress resistance than the wild type (WT) and the empty vector (UL) control. In transgenic plants, the activities of superoxide dismutase (SOD), peroxidase (POD), and catalase (CAT) were significantly enhanced; meanwhile, the contents of proline, malondialdehyde (MDA), and chlorophyll also changed significantly. In addition, by regulating the expression levels of *AtKIN1*, *AtCBF1*, *AtCBF2*, *AtCBF3*, *AtCOR47*, and *AtCOR15a*, *MbWRKY63* enhanced the low-temperature stress tolerance in transgenic *Arabidopsis*. The results suggest that *MbWRKY63* in apples may be involved in the response to low-temperature stress, laying a foundation for understanding the role of WRKY transcription factors (TFs) in abiotic stress responses.

## 1. Introduction

Plants face the dual challenges of biotic stress and abiotic stress during their growth process [1]. These can lead to the spread of plant diseases, inhibited growth, and even plant death [2,3]. In contrast, abiotic stresses are environmental factors that are not of biological origin, including low temperature, drought, salinity and alkalinity, heavy metal pollution, and ultraviolet radiation [4,5]. Stresses disrupt plants’ water balance, metabolic processes, and cell structures, harming agriculture and the environment [6]. To cope, plants respond to stress signals, transmit related information, and adjust physiologically/biochemically for adaptability-a process needing precise functional gene expression regulation. Among stressors, low temperature is common in northern abiotic environments [7,8]. Based on the degree of low temperature to which plants are exposed, low-temperature damage can be divided into two types: chilling damage (0–15 °C) and freezing damage (<0 °C) [9,10]. Under conditions of low-temperature stress, the activity of various enzymes in cells is inhibited, and electron transport in energy metabolism is blocked. This further leads to the excessive production of reactive oxygen radical species (ROS) [11,12]. At this point, the dynamic balance between the production and scavenging of ROS in plant cells is disrupted, triggering the massive accumulation of ROS in cells [13]. The accumulated ROS have strong oxidizing properties, which can directly oxidize amino acids to inhibit or destroy protein activity, and cause abnormal breakage of nucleic acids [14,15]. These effects induce abnormal cellular metabolic activities, such as augmented cell membrane permeability, electrolyte imbalance, membrane lipid peroxidation processes, and elevated levels of malondialdehyde (MDA) [16].

When facing biotic or abiotic stresses, plants first perceive and decode stress signals via a sophisticated transduction network (a series of molecular communications inside the cell), and subsequently trigger adaptive responses [17]. The response of plants to low-temperature stress is mainly mediated by the CBF (C-repeat binding factor) pathway and non-CBF pathways, in which low-temperature signals trigger downstream signaling cascades (sequences of molecular activations) [18]. In the CBF pathway, low temperature activates upstream regulatory factors, such as ICE1 (CBF Expression Inducer 1, a transcription factor), which promote the expression of the CBF TF family and trigger the synthesis of antifreeze proteins, dehydrins, and other substances to enhance the cell’s freezing resistance [19,20,21,22]. In contrast, non-CBF pathways function through a multi-signal network, including calcium signaling, ABA (Abscisic Acid) signaling pathways, and MAPK (Mitogen-Activated Protein Kinase) signaling [23,24,25]. These pathways work synergistically to activate the antioxidant system, scavenge ROS, reduce cell structure damage and metabolic disorders caused by low temperature, and collectively improve the plant’s low-temperature tolerance. In plants responding to abiotic stresses, signal cascades are mediated by numerous transcription factors, among which WRKY transcription factors (WRKY TFs) are an important group [26].

TF genes, also known as trans-acting elements, are genes whose transcripts and proteins derived from translation target the promoters of downstream genes, inhibiting or activating their transcription, thereby regulating gene expression and exerting their functions [27]. A large body of research has shown that members of transcription factor families, including WRKY, AP2/EREBP, bZIP, MYB, NAC, and TCP, play essential regulatory roles in plant responses to abiotic stresses [28]. WRKY TFs are a class of transcription factors found only in higher plants. Recent studies have confirmed that WRKY TFs can participate in various physiological and biochemical processes during plant growth and development [29]. For instance, they are involved in seed germination, root development, and flowering regulation, and can regulate hormone signal transduction. They also enhance the defense against biotic and abiotic stresses and maintain a balance between plant growth and environmental adaptability [30]. Current research indicates that studies on the role of WRKY transcription factors in plants’ response to abiotic stresses primarily focus on drought stress, while studies targeting low-temperature stress are relatively limited [31]. In recent years, studies have found that during the seed germination stage, WRKY proteins such as AtWRKY57 can bind to the W-box of *RD29A* and *NCED3* genes, endowing plants with drought tolerance and inhibiting seed germination [32]. Overexpression of *GmWRKY16*, *TaWRKY1*, or *TaWRKY33* in *Arabidopsis* can promote root growth and enhance drought tolerance under water stress conditions [33]. Additionally, many WRKY proteins have been confirmed to play a role in plant growth, developmental stages, and salt stress response processes. For example, overexpression of OsWRKY50 in rice can enhance the salt tolerance of transgenic plants [34]; MdWRKY100 in apples takes part in regulating the processes of salt stress responses in plants and is a downstream component of the miR156/SPL pathway [35]. These research results underscore the functions of WRKYs—both conserved and species-specific—in plant adaptation to abiotic stresses.

The WRKY family is one of the largest transcription factor families in higher plants [36]. The initial WRKY gene was isolated from sweet potato [37], and subsequently WRKY genes have been identified in various plant species [38]. WRKY proteins usually comprise one or two WRKY domains, each spanning approximately 60 amino acids. The N-terminal of these domains possesses the highly conserved “WRKYGQK” amino acid sequence [39]. During recent years, an expanding number of studies have confirmed the important role of WRKYs in plant responses to low-temperature stress. Overexpression of *SlWRKY46* positively regulates cold tolerance in tomato; *Arabidopsis*-based research has shown that *AtWRKY25* and *AtWRKY33* mediate cold and salt tolerance by modulating stress-responsive gene expression [40]. *AtWRKY63* performs a crucial role in plant responses to ABA and drought stress by regulating ABA-mediated stomatal closure and ABF2 expression [41]. There are 59 WRKY transcription factors in grapevine, 15 of which show obvious responses to chilling injury. In recent years, the rice transcription factors *OsWRKY71* and *OsWRKY76* have been identified as positive regulators of cold stress responses, enhancing cold tolerance, whereas *OsWRKY45* functions as a negative modulator, rendering rice hypersensitive to low-temperature stress [42]. However, the regulatory networks and mechanisms of WRKY TFs in low-temperature stress responses remain underexplored. Current research has mainly focused on plants such as soybean, rice, and wheat, while the molecular mechanisms underlying WRKY TF responses to low-temperature stress in horticultural crops, particularly woody plants like apple and grapevine, remain unclear [43].

*Malus baccata* (L.) Borkh is a fugacious arbor belonging to the genus *Malus* in the Rosaceae family. This tetraploid wild accession hybridizes with cultivated apple varieties, demonstrating excellent environmental adaptability and cold hardiness [44]. As a result, it acts as an essential germplasm resource for apple and apple rootstock breeding programs. During growth, *Malus baccata* is susceptible to abiotic stresses, including low temperature, drought, and salinity-alkalinity, which can reduce yield and quality. Nowadays, research on *Malus baccata* resources mainly focuses on the identification and assessment of stress-resistance-related phenotypic traits, lacking comprehensiveness and systematicity [45]. Therefore, molecular biology techniques should be integrated to study its stress resistance at the molecular level, aiming to clarify the stress regulatory mechanisms at the molecular scale and thereby facilitate better utilization of this germplasm in breeding programs [46]. Previous studies showed that *AtWRKY63* regulates vernalization and flowering, indirectly aiding low-temperature adaptation. Rice homolog *OsWRKY63* is cold-induced and has cold-responsive cis-elements (W-box, ABRE) in its promoter, directly confirming its role in low-temperature signaling. Despite WRKY63’s involvement in cold tolerance across species, the regulatory mechanism of *Malus baccata MbWRKY63* in cold stress remains unclear [47]. Thus, this study selected the *MbWRKY63* gene, which is potentially associated with low-temperature stress tolerance, for functional investigation. The *MbWRKY63* gene was cloned from *Malus baccata* and subjected to bioinformatics analysis. Real-time quantitative polymerase chain reaction (RT-qPCR) was used to determine the expression level of *MbWRKY63* in various *Malus baccata* tissues. In-depth exploration of its mechanism will reveal how the apple *MbWRKY63* transcription factor responds to low-temperature stress. This not only enriches the knowledge of the WRKY TF family in abiotic stress research but also provides a theoretical basis and genetic resources for the development of cold-resistant apple varieties, which is of great significance for improving apple stress tolerance and promoting the sustainable development of the apple industry [48].

## 2. Results

### 2.1. Cloning of the MbWRKY63 Gene and Corresponding Bioinformatics Analysis

**Figure 1:**
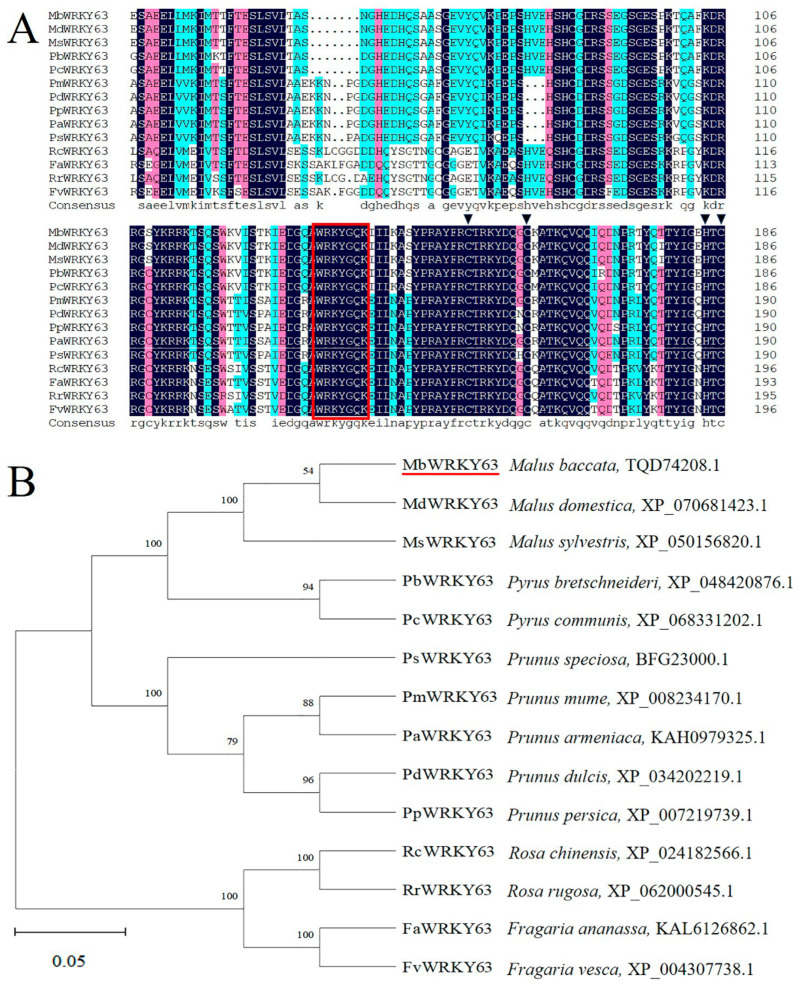
Amino acid sequence alignment between WRKY63 and sequences from other species, and phylogenetic tree construction. (**A**) Structure of the MbWRKY63 protein compared with WRKY63 proteins from different species. The red box indicates a WRKY conserved domain, and the black arrows mark the C_2_HC zinc finger motifs of WRKY. (**B**) Phylogenetic tree of WRKY63 proteins from different species and the MbWRKY63 protein. The content marked in red indicates the MbWRKY63 protein. These numbers represent the genetic relationships among other species, and the phylogenetic tree was constructed using MEGA7.0.

The WRKY TF *MbWRKY63* was isolated from *Malus baccata*, and its sequencing result is shown in Figure 1. The full length of *MbWRKY63* is 909 bp. Analysis via ExPASy-ProtParam revealed that the MbWRKY63 protein consists of 302 amino acids (Figure S1), among which serine (Ser, S) accounts for 11.9%, glutamic acid (Glu, E) for 7.3%, lysine (Lys, K) for 7.3%, glycine (Gly, G) for 6.6%, and serine (Ser, S) for 6.6%, being the most abundant amino acids. The molecular weight (MW) of MbWRKY63 is 33,960.82 kDa, with a theoretical isoelectric point (pI) of 6.38; its grand average of hydropathicity (GRAVY) is −0.783, indicating that it is a hydrophilic protein; the instability index is 49.23, suggesting that this protein is unstable.

The sequence analysis showed that the MbWRKY63 protein contains only one conserved WRKY domain and a C_2_HC zinc finger structure; thus, this protein belongs to the class III WRKY family (Figure 1A). In this study, BLAST was used to align the MbWRKY63 protein sequence to homologs. It was determined that MdWRKY63 from *Malus domestica* (XP_070681423.1), MsWRKY63 from *Malus sylvestris* (XP_050156820.1), PbWRKY63 from *Pyrus bretschneideri* (XP_048420876.1), PcWRKY63 from *Pyrus communis* (XP_068331202.1), PsWRKY63 from *Prunus speciosa* (BFG23000.1), PmWRKY63 from *Prunus mume* (XP_008234170.1), PaWRKY63 from *Prunus armeniaca* (KAH0979325.1), PdWRKY63 from *Prunus dulcis* (XP_034202219.1), PpWRKY63 from *Prunus persica* (XP_007219739.1), RcWRKY63 from *Rosa chinensis* (XP_024182566.1), RrWRKY63 from *Rosa rugosa* (XP_062000545.1), FaWRKY63 from *Fragaria ananassa* (KAL6126862.1), and FvWRKY63 from *Fragaria vesca* (XP_004307738.1) all show high homology with MbWRKY63. They share the same WRKYGKK sequence and C_2_HC zinc finger motif (Figure 1B). These results suggest that MbWRKY63 belongs to the WRKY TF family. Phylogenetic analysis shows that MbWRKY63 and MdWRKY63 are more closely related.

**Figure 2:**
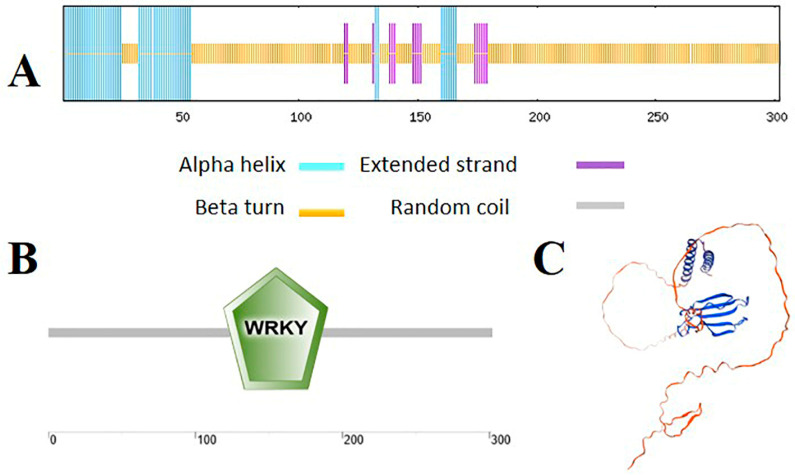
Prediction of the MbWRKY63 protein’s secondary and tertiary structures (**A**) Analysis of the secondary structure of the MbWRKY63 protein. (**B**) Investigation into the conserved structural domain of the MbWRKY63 protein. (**C**) Forecasting of the tertiary structure of the MbWRKY63 protein.

Using SOPMA, we predicted the secondary structure of the protein encoded by the *MbWRKY63* gene, revealing that it consists of 21.91% α-helix, 70.99% random coil, and 7.10% extended strand (Figure 2A). SMART prediction showed that MbWRKY63 contains a WRKY domain at amino acid positions 126–188, indicating that it belongs to the WRKY family and harbors conserved WRKY domains (Figure 2B). In addition, its tertiary structure was predicted by SWISS-MODEL, which is consistent with the secondary structure prediction and similar to that of other proteins in the WRKY family (Figure 2C).

### 2.2. Subcellular Localization of the MbWRKY63 Gene in the Nucleus

For the investigation of the *MbWRKY63* gene’s subcellular localization, since *MbWRKY63* acts as a transcription factor, the transient expression fusion vector *MbWRKY63*-pCAMBIA1300 was developed for tobacco-related studies, with 35S::GFP serving as the control. The constructed transient overexpression vector was transformed into *Agrobacterium*, and the *Agrobacterium* was then injected into the outer epidermal cells of *Nicotiana benthamiana* leaves to conduct the subcellular localization experiment.

**Figure 3:**
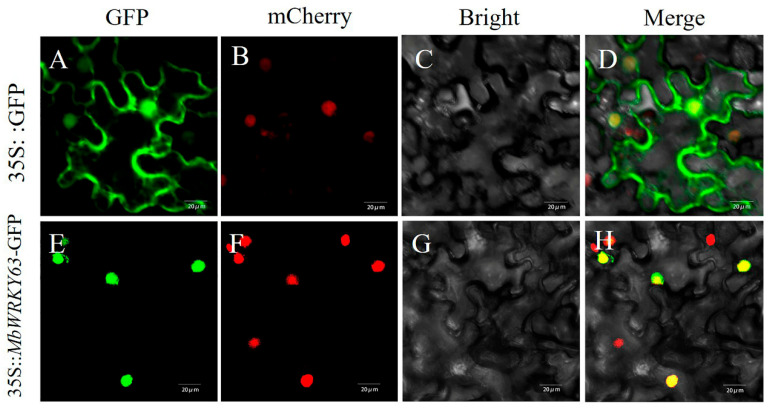
Subcellular localization characteristics of the MbWRKY63 protein. Transient expression assays of 35S::*MbWRKY63*::GFP were performed in tobacco leaves, with 35Spro::GFP designated as the positive control. (**A**,**E**) GFP signals; (**B**,**F**) mCherry signals; (**C**,**G**) Bright field views; (**D**,**H**) Merge views. Since mCherry serves as a nuclear marker, the yellow color in the merged images indicates that GFP and mCherry are colocalized. The scale bars correspond to 20 µm.

As shown in Figure 3, using a laser confocal microscope, the distribution of green fluorescent protein (GFP) in *Nicotiana benthamiana* leaves of the control group was observed: fluorescent signals were detected in both the plasma membrane and the nucleus. In contrast, the green fluorescence of 35S::*MbWRKY63*::GFP was only observed in the nucleus, as also illustrated in Figure 3. Additionally, observation of red fluorescence from the nucleus confirmed that MbWRKY63 is a nuclear-localized protein.

### 2.3. Analysis of MbWRKY63 Gene Expression Level in Malus baccata

**Figure 4:**
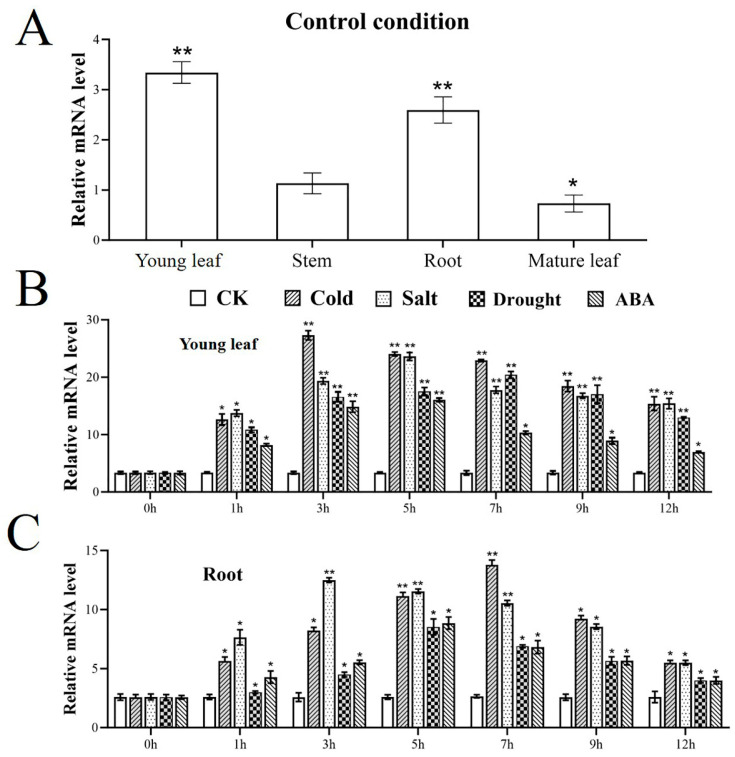
Analysis of tissue-specific and stress-responsive expression profiles of *MbWRKY63* in *Malus baccata.* (**A**) Different degrees of *MbWRKY63* expression are observed in young leaves, stems, roots, and mature leaves. (**B**) Time-course of *MbWRKY63* expression in young leaves in control and under low-temperature (4 °C), salt (200 mM NaCl), dehydration (6% PEG6000), and abscisic acid (50 µM) treatments. (**C**) Temporal expression pattern of *MbWRKY63* in young leaves under control conditions and treatments with low-temperature (4 °C), Salt (200 mM NaCl), dehydration (6% PEG6000), and ABA (50 µM). Error bars represent the standard deviation, and asterisks located above the error bars indicate that there are significant differences between the treatment and control groups (Student’s *t*-test; * *p* ≤ 0.05, ** *p* ≤ 0.01).

Analysis of the tissue-specific expression pattern of *MbWRKY63* across different organs of *Malus* seedlings, including young leaves, stems, roots, and mature leaves, was examined using qRT-PCR (quantitative real-time polymerase chain reaction). The results showed that *MbWRKY63* exhibited relatively elevated expression in young leaves and roots, whereas its expression was low in stems and mature leaves. This suggests that the primary function of *MbWRKY63* may be related to nutrient uptake and transport (Figure 4A).

*MbWRKY63* expression could be induced under different stress conditions, including 4 °C (low temperature), 200 mM NaCl (high salt), 6% PEG6000 (drought mimic), and 100 µM ABA. Within 12 h of stress treatment, the expression level of *MbWRKY63* under various stresses showed a single-peak distribution pattern. The expression of *MbWRKY63* in young leaves and roots reached peak values at multiple time intervals, with comparable peak levels. As shown in Figure 4B,C, *MbWRKY63* showed greater sensitivity to three stresses: low-temperature, high-salt, and drought.

Under these four applied stress conditions (low temperature, high salt, drought, and ABA), the expression level of *MbWRKY63* in young leaves first increased and then decreased. Within young leaf tissues, the peak expression times of *MbWRKY63* under different abiotic stresses were 3 h (low temperature), 5 h (high salt), 7 h (drought), and 5 h (ABA). Within root tissues, the relative expression level of *MbWRKY63* reached its peak at 3 h, 7 h, 5 h, and 5 h under the aforementioned abiotic stresses, respectively. After 3 h of 4 °C treatment, the peak expression level of *MbWRKY63* in young leaves was 8.139 times that of the control group; after 7 h of 4 °C treatment, the peak expression level of *MbWRKY63* in roots was 5.19 times that of the control group (Figure 4B,C). It indicates that low-temperature stress can efficiently induce upregulation of *MbWRKY63* in various tissues. Furthermore, a comparison of low-temperature stress with other abiotic stresses (high salt, drought) and hormonal stress (ABA) shows that only low-temperature stress results in a significantly greater upregulation of *MbWRKY63* expression than the other treatments. Moreover, this upregulation exhibits distinct tissue specificity and time dependence (an early peak in young leaves and a late peak in roots). This indicates that *MbWRKY63* displayed extreme sensitivity toward low-temperature stress.

### 2.4. Overexpression of MbWRKY63 Enhances Cold Stress Tolerance in Arabidopsis thaliana

**Figure 5:**
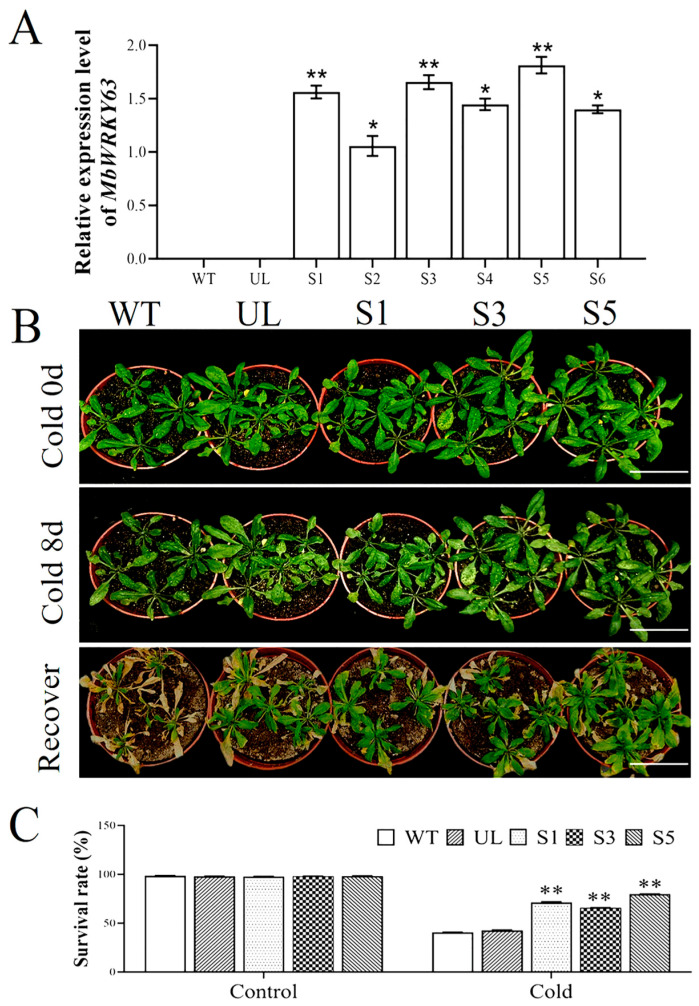
The *MbWRKY63* gene confers cold tolerance in *Arabidopsis*. (**A**) Expression of *MbWRKY63* in transgenic lines S1–S6, UL, and WT; (**B**) Phenotypes of transgenic high-expression lines (S1, S3, S5), UL, and WT. Scale bar is 3 cm; (**C**) Survival rate of each *Arabidopsis* line under cold stress (4 °C). Asterisks above indicate statistically significant differences compared with WT (* *p* ≤ 0.05; ** *p* < 0.01).

To investigate whether *MbWRKY63* exerts a regulatory role in the cold stress tolerance of *Arabidopsis thaliana*, a fusion vector *MbWRKY63*-pCAMBIA1300 was constructed. The pCAMBIA1300 empty vector and *MbWRKY63*-pCAMBIA1300 vector were transformed into *Arabidopsis thaliana* to obtain transgenic plants. Kanamycin resistance-based positive screening for overexpressed *MbWRKY63* was performed in T1 transgenic *Arabidopsis* lines and empty vector control lines (UL). Total RNA was extracted from positive plants, and RT-qPCR was performed. Using wild-type (WT) and empty vector (UL) plants as control materials, no *MbWRKY63* expression was detected in these control groups. At the same time, *MbWRKY63* was expressed at varying levels in the T2 generation of positive transgenic *Arabidopsis*. This result indicated that *MbWRKY63* was successfully transferred into *Arabidopsis thaliana* (Figure 5A). Leaf-derived genomic DNA was extracted from six transgenic *Arabidopsis* lines (S1, S2, S3, S4, S5, and S6) and verified by specific PCR (primers: *MbWRKY63*-F/R, Table S1). The results showed that all transgenic lines exhibited the target band, which was absent in WT and UL plants. Among these lines, the expression levels of S1, S3, and S5 were significantly higher than those of S2, S4, and S6 (Figure 5A). Three lines with high *MbWRKY63* expression were selected and carefully cultured to the T3 generation. Subsequently, for each plant line, 50 plants (5 per pot) were selected to calculate the survival rate. Abiotic stress treatment was applied to WT, UL, and transgenic plants (S1, S3, S5). Under non-stress conditions (control, 4 °C), the growth trends of *Arabidopsis* lines were generally consistent, with no significant variation in survival rate observed. After 8 days of cold treatment at 4 °C, the leaves of both overexpressing plants (S1, S3, S5) and control plants (WT, UL) turned yellow, showing obvious phenotypic differences, and all plants exhibited varying degrees of wilting. However, the leaves of transgenic *Arabidopsis* lines overexpressing *MbWRKY63* (S1, S3, S5) remained green (Figure 5B). After 3 days of recovery under normal growth conditions, the survival rates of seedlings from transgenic lines S1, S3, and S5 were 75%, 69%, and 82%, respectively. In contrast, the survival percentages of WT and UL plants were 38% and 40%, respectively, and their leaves were severely affected, showing wilting and yellowing (Figure 5C). These experimental results demonstrated that *Arabidopsis thaliana* overexpressing *MbWRKY63* exhibited a higher survival rate under cold stress conditions than WT and UL plants.

**Figure 6:**
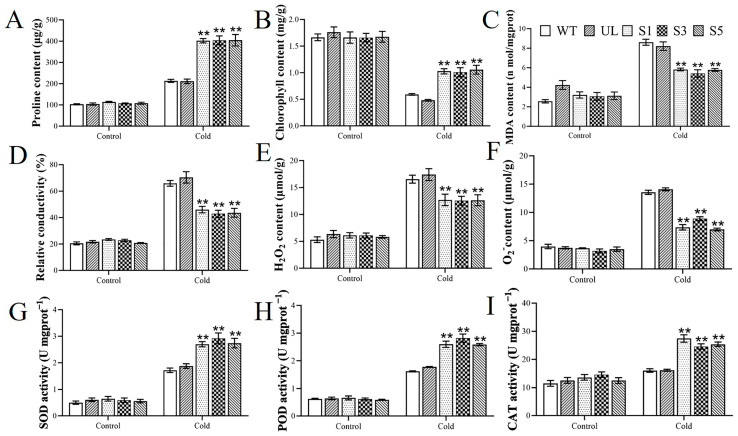
Impacts of *MbWRKY63* on stress-responsive physiological indices in *Arabidopsis* under low-temperature (4 °C) stress. Contents of (**A**) proline, (**B**) chlorophyll, (**C**) MDA, (**D**) Relative conductivity, (**E**) H_2_O_2_, (**F**) O_2_^−^, and the active of (**G**) SOD (**H**) POD (**I**) CAT in the WT, UL, and *MbWRKY63*-OE lines (S1, S3, and S5) under 4 °C treatment for 8 days. For each group, the mean of three replicates is shown with the standard error, and asterisks above the error bars highlight significant differences between transgenic and WT lines (Student’s *t*-test, ** *p* ≤ 0.01).

Under the control condition (4 °C for 0 days), the stress resistance-related physiological indexes of various *Arabidopsis thaliana* lines showed no significant differences, including chlorophyll, proline, superoxide dismutase (SOD), peroxidase (POD), and catalase (CAT). However, under cold stress at 4 °C, changes occurred in all physiological indices: the proline content and chlorophyll content increased in overexpressing plants, and the activities of antioxidant enzymes (SOD, POD, CAT) were enhanced (Figure 6A,B,G–I). In contrast, the contents of MDA, H_2_O_2_, O_2_^−^, along with relative electrical conductivity, were lower in *Arabidopsis thaliana* lines that overexpress *MbWRKY63* (Figure 6C–F). This indicates that the *Arabidopsis thaliana* overexpressing *MbWRKY63* suffered less damage under abiotic stress (Figure 6). The determination of plant physiological indices before and after cold stress further demonstrated that overexpression of *MbWRKY63* enhanced cold tolerance and significantly improved the plants’ response to extreme cold.

### 2.5. MbWRKY63 Activates Cold Stress-Tolerance-Related Downstream Genes in Arabidopsis thaliana

**Figure 7:**
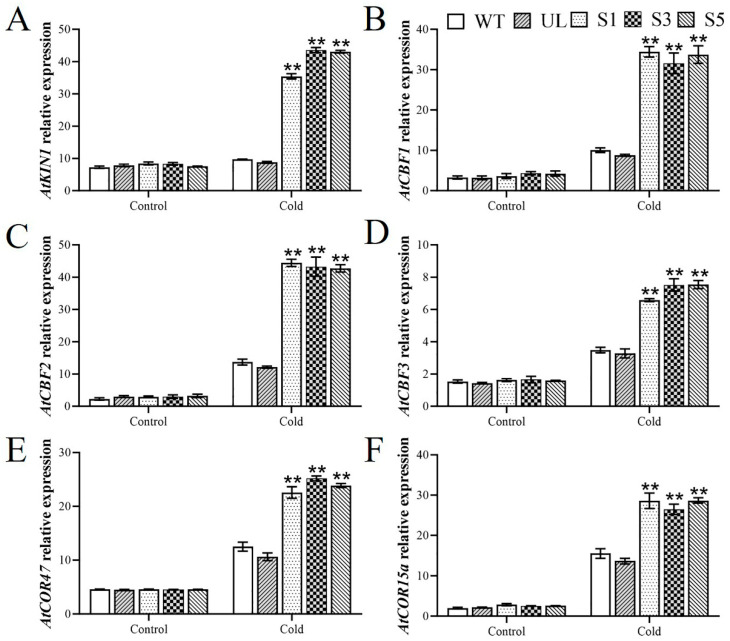
Impacts of *MbWRKY63* on the expression of cold tolerance-associated genes in transgenic *Arabidopsis* under low-temperature stress (**A**) *AtKIN1*; (**B**) *AtCBF1*; (**C**) *AtCBF2*; (**D**) *AtCBF3*; (**E**) *AtCOR47* and (**F**) *AtCOR15a*. The standard error accompanies the mean value of three replicate experiments, and asterisks positioned above the error bars indicate significant differences between transgenic lines and WT lines (Student’s *t*-test, ** *p* ≤ 0.01).

To further clarify the regulatory mechanism of the *MbWRKY63* gene under cold stress, the expression levels of downstream related genes, including *AtKIN1* (AT3G63480), *AtCBF1* (AT4G25490), *AtCBF2* (AT4G25470), *AtCBF3* (AT4G25480), *AtCOR47* (AT1G20440), and *AtCOR15a* (AT2G42540), were analyzed via qRT-PCR in this experiment. Under cold stress conditions, analysis of the expression patterns of these genes indicated that their expression in *MbWRKY63*-overexpressing lines was significantly higher than that in the wild-type (WT) and empty vector (UL) lines (Figure 7A–F). These findings demonstrate that the overexpression of *MbWRKY63* may induce the expression of downstream cold-stress tolerance-related genes, thereby enabling these genes to exert functions such as participating in the synthesis of membrane-related substances, maintaining intracellular osmotic balance, and preventing membrane lipid peroxidation. Ultimately, this enhances the cold stress tolerance of transgenic *Arabidopsis thaliana*.

## 3. Discussion

**Figure 8:**
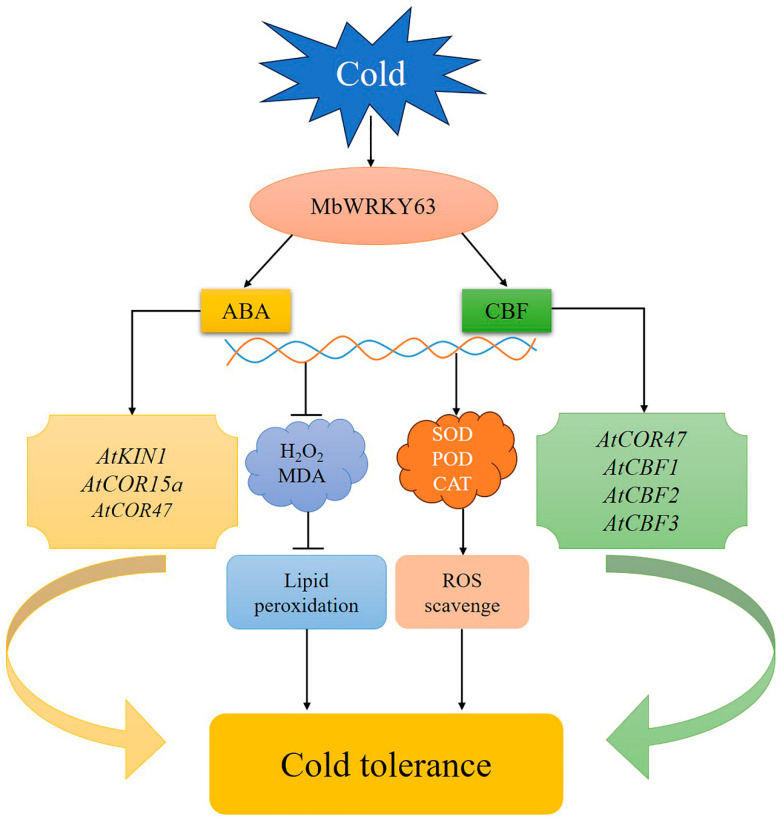
Schematic diagram of the possible molecular mechanism of *MbWRKY63* in plant cold stress tolerance. The activated *MbWRKY63* can significantly alleviate cell damage caused by ROS and lipid peroxides, and promote the increased expression of downstream stress-associated genes. Under low-temperature conditions, plants are exposed to signals that activate *MbWRKY63*. On the one hand, *MbWRKY63* contributes to the ABA signaling pathway and promotes the expression of downstream genes (*AtKIN1*, *AtCOR47*, and *AtCOR15a*), thereby enhancing plant tolerance to cold environments. On the other hand, *MbWRKY63* regulates CBF cold-regulated genes through an ABA-independent signaling pathway to activate *COR47* expression; it also participates in the CBF pathway, promotes the expression of downstream *AtCBF1*, *AtCBF2*, and *AtCBF3* genes, and ultimately enhances plant cold tolerance.

WRKY transcription factors play a crucial role in regulating plant responses to cold or heat stress. Studies have shown that WRKYs respond to extreme temperature stress by regulating ABA-responsive gene expression. Under cold stress, the relative expression of ABI5 is upregulated in transgenic *Arabidopsis thaliana* plants overexpressing *ClWRKY20* from watermelon (*Citrullus lanatus*), resulting in a significant enhancement of cold stress tolerance [49]. Under cold stress, WRKY53 binds to the promoters of genes involved in gibberellin (GA) synthesis and inhibits their expression, thereby reducing rice’s cold tolerance [50]. In *Arabidopsis thaliana*, overexpression of *PmWRKY57* or *KoWRKY40* from *Kandelia obovata* (a mangrove species) increases the expression levels of genes responsive to cold stress (*AtCOR6.6* and *AtCOR47*, respectively) and antioxidant enzyme-related gene (*AtMnSOD*), which in turn enhances the cold stress tolerance of *Arabidopsis thaliana* [51,52]. Although research on the WRKY TF family in other model organisms is relatively comprehensive, studies on the cold stress response mechanism controlled by WRKY TFs in the apple cultivar *Malus baccata* remain relatively scarce (Figure 8).

In this study, we isolated and identified a cold-stress-induced gene from the apple cultivar *Malus baccata*. This gene contains a highly conserved WRKY domain (Figure 2B), which shares high similarity with the WRKY63 domains from other species; therefore, we named it *MbWRKY63*. Structural and phylogenetic analyses of this gene revealed that its full-length sequence is 909 base pairs (bp) and encodes 302 amino acids. Genes of the WRKY TF family all possess a conserved WRKYGQK sequence and a zinc finger motif (C_2_HC) in their DNA-binding domains, which can interact with the W-box (TTGAC) cis-acting element. In our study, we found that the amino acid sequence of MbWRKY63 harbors a WRKYGKK motif and a C_2_HC zinc finger domain (Figure S1). Studies on protein properties showed that the MbWRKY63 protein has a theoretical molecular weight (MW) of 33,960.82 kDa, a theoretical isoelectric point (pI) of 6.38, and a grand average of hydropathicity (GRAVY) value of −0.783, indicating it is a hydrophilic protein. Additionally, its instability index is 49.23, classifying it as an unstable protein. Phylogenetic tree analysis of the MbWRKY63 protein was performed using MEGA7.0. The results indicated that it shares conserved nucleotide similarity with WRKY proteins from other species (Figure 1A) and shares the closest genetic affinity with MdWRKY63 (Figure 1B). Structural analysis of the MbWRKY63 protein showed that it contains the conserved characteristic domain of the WRKY family (Figure 2A). Its tertiary structure (Figure 2C) harbors a conserved WRKY domain, and MbWRKY63 belongs to the WRKY group III.

Numerous studies have investigated the subcellular localization of WRKY TFs, and the results have shown that fluorescent signals are exclusively detected in the nucleus [53]. To accurately determine the intracellular localization of the MbWRKY63 protein, identify its functional domains, and gain insight into its mechanism of action, the constructed transient expression fusion vector *MbWRKY63*-pCAMBIA1300 was introduced into *Nicotiana benthamiana* leaf epidermal cells using *Nicotiana benthamiana* transient transformation. We found that its nuclear localization (Figure 3) is consistent with its role as a transcription factor [54]. Gene expression profiles are usually closely associated with gene function. For instance, in roots, stems, leaves, flowers, and fruits, *MdWRKY53* exhibits expression, with the highest expression level in fruits and the lowest in stems [55]. Multiple studies have demonstrated that WRKY genes exhibit tissue-specific expression throughout development, reflected by differences in gene expression levels across various tissues and organs [56]. Therefore, in this experiment, we analyzed transcriptional expression of *MbWRKY63* in the root system, stem tissues, young leaves, and old leaves of the apple cultivar *Malus baccata*. As shown in Figure 4, the *MbWRKY63* gene showed the maximum expression level in young leaves and the lowest in old leaves, suggesting that this gene exhibits greater sensitivity to abiotic stress in newly developed organs. The expression of WRKY genes is regulated by plant hormones and induced by abiotic stresses alike [57,58]. For example, in apple, *MdWRKY55* interacts with MdNAC17-L and activates the *MdNHX1* expression, thereby enhancing salt tolerance [59]. *MdWRKY35* in apple is induced by four stimuli (ABA, drought, salt, cold stress), while *MdWRKY53* is involved in cold, salt stress, and four hormone signaling pathways (ABA, IAA, GA, SA) [60]. Under different stress inductions, gene expression also varies depending on their functions.

The research findings regarding the expression profile and functional properties of *MbWRKY63* in *Malus baccata* are consistent with previous reports in other plant species and extend them further. For example, in *Arabidopsis thaliana*, *AtWRKY34* and *AtWRKY63* mediate vernalization by regulating FLC expression, promote cold-induced flowering, and modulate plant stress responses to stimuli such as ABA and drought. Additional functional verification in transgenic *Arabidopsis* emphasizes the conserved function of WRKY TFs in abiotic stress tolerance. Although overexpression of *MbWRKY63* did not alter the normal growth phenotype, compared with the wild type, the survival rate of transgenic lines increased by 45% at 4 °C (Figure 5C). Seeds of *Arabidopsis* overexpressing *MbWRKY63* exhibited higher survival rates and stronger growth ability under cold stress. No pleiotropic or unintended effects were detected, indicating that the overexpression of *MbWRKY63* exhibits good application safety in *Arabidopsis*. These results indicate that *MbWRKY63* may participate in responses to various stresses and exhibit differences in its regulatory mechanisms across different stress response pathways, thereby enhancing apple tolerance to low-temperature osmotic stress.

Excessive reactive oxygen species (ROS) can cause severe damage to plant proteins [61]. Cold and drought stress can generate two major types of ROS: hydrogen peroxide (H_2_O_2_) and superoxide anion (O_2_^−^) [62,63]. Changes in H_2_O_2_ content can act as intracellular signals, mediating oxidative stress responses in plants by triggering the expression of multiple antioxidant enzyme genes and thereby increasing antioxidant enzyme activity. An antioxidant system exists in plant cells, consisting of various antioxidant molecules and antioxidant enzymes-SOD, CAT, POD, ascorbate peroxidase (APX), guaiacol peroxidase (GPX), and glutathione reductase (GR) included. This system maintains ROS at levels that do not interfere with normal cellular functions and plays a dominant role in reducing ROS-induced cell damage and maintaining ROS homeostasis [64]. In this study, a comparative analysis indicated that *Arabidopsis thaliana* plants overexpressing *MbWRKY63* exhibited stronger cold tolerance than the control group. *MbWRKY63* may be associated with reduced lipid peroxidation, membrane and cellular damage [65,66], accompanied by increased antioxidant enzyme activity and decreased ROS accumulation. Multiple lines of evidence indicate that overexpression of *MbWRKY63* enhances the activity of the antioxidant enzyme system under stress conditions and positively regulates ROS scavenging. Thus, plant membrane damage triggered by ROS can be lessened, while plants’ tolerance to cold and drought can be enhanced (Figure 5 and Figure 6). MDA can be used as an indirect indicator to predict plant stress tolerance [67]. Exposure to stress can damage the structure and function of cell membranes, ultimately leading to electrolyte leakage and increased permeability. EL serves as an important indicator reflecting stress-induced damage to plant cell membranes [68]. Plants overexpressing *MbWRKY63* suffered less membrane damage under stress, and their EL and MDA contents were lower compared with the control group. When exposed to cold stress, plants can produce osmolytes, such as soluble sugars and proline, which help scavenge excess ROS and reduce stress-induced damage [69]. Proline helps sustain cellular osmotic balance, scavenges hydroxyl radicals, and strengthens plant stress tolerance [70]. Compared with the WT and UL, *Arabidopsis thaliana* overexpressing *MbWRKY63* showed higher proline content and stronger resistance to adverse environments, enabling it to maintain chlorophyll stability and photosynthetic capacity. As the most important process governing plant productivity, photosynthesis relies on chlorophyll, a key component (Figure 6).

WRKY TFs serve as key nodes in the ABA-responsive signaling pathway network. Studies have found that WRKY gene expression activates downstream genes with functional roles (*KIN1*, *RD29A*, and *COR47*) that are engaged in cold and drought stress responses through an ABA-dependent pathway, thereby enhancing the cold and drought tolerance of transgenic plants. Currently, studies on *Malus gyllenhalii* have shown that *KIN1*, a key factor, is involved in the plant’s cold stress response [71]. Numerous studies have reported that *COR47* plays a positive regulatory role in plant cold stress resistance. CBF proteins, on the other hand, are important for mediating abiotic stress response [72]. Studies in different plant species have confirmed that CBF genes respond to cold, drought, and other stress treatments [73,74,75]. By binding to the CRT (C-repeat) cis-acting element in the *COR47* promoter, the cold-positive regulator CBF activates *COR47* expression, which ultimately enhances plant cold tolerance [76]. Drought stress induces ABA accumulation, which enables CBF proteins to interact with CRT/DRE (dehydration-responsive element) elements and trigger the expression of their target genes [77,78]. In *WRKY46*-overexpressing *Arabidopsis*, the ABA-independent pathway-related genes CBF1, CBF2, and CBF3 were identified. The three CBF TFs bind to the C-repeat/dehydration-responsive element (CRT/DRE) DNA regulatory elements located in the promoters of cold-regulated (COR) genes. Cold stress upregulates CBF expression, and overexpression of CBFs confers enhanced constitutive freezing tolerance to plants [79]. To analyze the potential downstream functional genes through which *MbWRKY63* may act in response to cold stress, we examined the expression levels of key genes involved in the cold stress response pathway in transgenic plants after cold stress exposure (Figure 7) [80]. The results revealed that the ABA pathway contributes to the enhanced functions of *KIN1*, *COR47*, and *COR15a*. Several studies have shown that the *DREB2A* gene specifically binds to DRE cis-acting elements, regulates the expression of a series of stress-responsive genes under drought and cold stress conditions, and improves plants’ overall tolerance to stress [81]. Our findings reveal that *MbWRKY63* expression is induced by cold stress in *M. baccata*. Compared with the WT and UL lines, the transcriptional levels of these six genes were increased in *MbWRKY63*-overexpressing lines. This indicates that *MbWRKY63* can directly or indirectly regulate the expression of *AtKIN1*, *AtCOR47*, *AtCOR15a*, *AtCBF1*, *AtCBF2*, and *AtCBF3*, thereby enhancing cold stress tolerance (Figure 8).

## 4. Materials and Methods

### 4.1. Tested Plant Materials, Cultivation Conditions, and Treatments

Seeds of *Malus baccata* were collected from mature and plump fruits of crabapple trees located in front of the Building of College of Horticulture and Landscape Architecture, Northeast Agricultural University. Murashige and Skoog (MS) medium was used for sowing *M. baccata* seeds (Coolaber, PM1011, Beijing, China), and the seedlings were then transferred to a medium containing 0.6 mg/L indole-3-butyric acid (IBA) and 0.6 mg/L 6-benzylaminopurine (6-BA). Finally, seedlings growing toward the MS medium were selected, and at this stage, the medium was modified to contain 1.2 mg/L IBA. After the seedlings developed roots, they were shifted to the Hoagland nutrient solution for acclimatization culture. The culture conditions were maintained at 25 °C with a relative humidity of 80 ± 5%. The hydroponic solution was renewed every 3–4 days, and the seedlings were cultured for 50 days. Once the seedlings had 7–9 fully expanded true leaves and a robust root system, they were evenly split into 5 groups [82]. One group was used for sampling roots, stems, young leaves, and old leaves; the remaining 4 groups were subjected to stress treatments, with one of these 4 groups designated for collecting root, stem, young leaf, and old leaf samples, and the other 4 groups subjected to the following 4 stress treatments: (1) Cold stress treatment: 4 °C light incubator was used to culture hydroponic seedlings; (2) Salt stress treatment: 200 mM NaCl high-salt medium was used to culture hydroponic seedlings; (3) Drought stress treatment: Hoagland nutrient solution (6% PEG6000) was used for hydroponic seedlings; (4) ABA stress treatment: Hoagland medium (50 μM ABA) was used for hydroponic seedlings. Young leaf and root samples of *M. baccata* were collected at 0 h, 1 h, 3 h, 5 h, 7 h, 9 h, and 12 h after the initiation of the 4 treatments. The same tissues (young leaves, old leaves, roots, and stems) were collected from 5 uniform-growth seedlings, mixed, and used as a single sample (fresh weight approximately 100–150 mg). This approach reduces the interference of growth differences between individual plants on the detection results. Samples were immersed in liquid nitrogen immediately after collection, then stored at −80 °C for RNA extraction [83].

An appropriate amount of *Arabidopsis thaliana* seeds was soaked in sterile distilled water and incubated overnight at ambient conditions. Subsequently, the seeds were sterilized using aseptic techniques on a clean bench. The *Arabidopsis thaliana* seeds were placed in a 1.5 mL centrifuge tube. First, they were soaked in 75% ethanol for 30 s, followed by rinsing with sterile distilled water 4–5 times to remove residual ethanol. Then, the seeds were mixed with a 5% sodium hypochlorite solution and soaked for approximately 10 min. Last, sterile distilled water wash (4–5 times) to remove residual sodium hypochlorite. The sterilized seeds were sown on MS-based seed medium, and the seeds were spread evenly to ensure maximum growth space. After being placed at 4 °C for 2–3 days, the medium was transferred to a tissue culture room. When the seeds germinated, developed roots, and formed 2–4 cotyledons, the *Arabidopsis thaliana* seedlings were transplanted into nutrient-rich soil (peat moss/vermiculite = 2:1) and cultured as described by Cutler et al. [84].

### 4.2. Isolation and Cloning of MbWRKY63

*Malus* seedling tissues (young leaves, roots, stems, mature leaves): total RNA extracted with plant RNA kit (Sangon Biotech, B518661-0100, Shanghai, China), following the instructions provided in the kit. First-strand cDNA: synthesized with reverse transcription kit (TransGen Biotech, Beijing, China). The nucleotide sequence of the reference gene *MbWRKY63* (Accession No.: TQD74208.1) was obtained from NCBI (https://www.ncbi.nlm.nih.gov, accessed on 5 December 2025). Specific primers (*MbWRKY63*-F/R; Table S1) were designed using Primer 5.0 software, with the coding sequence (CDS) region of this gene as the reference. The target gene was amplified using a PCR instrument, with the synthesized first-strand cDNA as the template. The PCR conditions were as follows: Pre-denaturation: 94 °C/2 min; 34 cycles (94 °C/30 s, 55 °C/30 s, 72 °C/30 s); final extension: 72 °C/2 min. DNA purification kit used to recover PCR product (CWBIO, CW2301M, Nanjing, China). Subsequently, the target fragment was ligated into the pEASY-T5 Zero vector (TransGen, Beijing, China); the DNA fragment was gel-purified and then sequenced (BGI) [85].

### 4.3. Bioinformatics Analysis of the MbWRKY63 Gene

Post-acquisition of *MbWRKY63* gene sequencing data, sequence alignment was performed using EMBOSS (http://www.ebi.ac.uk/Tools/psa/emboss_needle/, accessed on 5 December 2024). The nucleotide sequence of the gene was translated using DNAMAN 5.2 software. A NCBI-BLASTp search of the amino acid sequence of MbWRKY63 was conducted on NCBI (https://blast.ncbi.nlm.nih.gov/Blast.cgi, accessed on 4 January 2025). Thirteen amino acid sequences from other species with high homology to MbWRKY63 were selected for homology analysis with MbWRKY63, and a phylogenetic tree was constructed using MEGA7.0 (http://www.megasoftware.net, accessed on 4 January 2025). In addition, the basic structure of the MbWRKY63 protein was predicted using ExPASy (https://web.expasy.org/protparam/, accessed on 17 February 2025); Prediction of the protein’s secondary structure was conducted using SOPMA (https://npsa-prabi.ibcp.fr/, accessed on 18 February 2025); its tertiary structure was predicted via SWISS-MODEL (https://swissmodel.expasy.org/, accessed 20 February 2025); and the domains of the MbWRKY63 protein were predicted using SMART (http://smart.embl-heidelberg.de/, accessed on 21 February 2025).

### 4.4. Subcellular Localization of the MbWRKY63 Protein

Restriction endonucleases *BamH* I and *Xba* I were used to linearize the 35S-sGFP-pCAMBIA1300 vector. Specific primers with restriction enzyme sites (*MbWRKY63*-sl F/sl R; Table S1) were designed, and the gene fragment containing restriction enzyme sites was amplified via PCR [86]. Double digestion was performed on both the PCR product and the 35S-sGFP-pCAMBIA1300 vector. Ligation of the target fragment with the vector yielded the *MbWRKY63*-GFP transient expression vector. As a typical WRKY family transcription factor, the N-terminus of *MbWRKY63* is its functional core region, which mainly contains the “WRKYGQK” sequence and the C_2_HC-type zinc finger structure. GFP was fused to the N-terminus of *MbWRKY63* to enable accurate protein localization and verify its function. Using a mCherry-containing plasmid as a template, the mCherry coding region was PCR-amplified with primers containing vector-matching restriction sites. The mCherry fragment and the vector backbone were double-digested and ligated [87,88]. Last, *Agrobacterium*-mediated transformation was performed, and the transformed *Agrobacterium* was resuspended in a buffer solution (10 mM MgCl_2_ + 10 mM MES + 200 μM acetosyringone). Adjust the OD to 0.4, and store the suspension at ambient conditions for 2–3 h. The inoculum was infiltrated into the expanded leaves of 5–6-week-old *Nicotiana benthamiana*. After 2 days of low-light culture, leaves were collected; stained leaves were observed via laser confocal microscope to determine the *MbWRKY63*-encoded protein’s intracellular localization [89].

### 4.5. RT-qPCR Analysis of the MbWRKY63 Gene

As reported in previous studies [90], *MbWRKY63* was detected in *Malus* seedling roots, stems, young, and mature leaves. Different plant parts underwent stress treatments (low temperature, high salt, drought, ABA) (Solarbio, A8060, Beijing, China), and the expression of *MbWRKY63* after treatment was examined. Specific primers for qPCR (*MbWRKY63*-qF/qR; Table S1) were designed based on the conserved region of *MbWRKY63*. The standardized internal reference gene *Mbactin* and its primers (*Mbactin*-F/R; Table S1) were used. The treated plant materials were used as templates for cDNA synthesis. Components of the real-time PCR detection reagent TBlazeTaq™ SYBR^®^ Green qPCR Mix 2.0 (Cat. No.: QP031, Guangzhou, China) were added. RT-qPCR analysis of the *MbWRKY63* gene was performed using a Bio-Rad (CFX 96, Hercules, CA, USA) Real-Time PCR System. The collected data were analyzed using the 2^−ΔΔCT^ method [91].

### 4.6. Acquisition of Transgenic Plants

Based on the principle of homologous recombination, an overexpression vector was constructed using Primer 5.0 software. Vector primers (designed) included homologous sequences, restriction sites, and specific amplification primers (*MbWRKY63*-F/R; Table S1). The 5′ end (*BamH* I restriction site) and 3′ end (*Xba* I restriction site) of the *MbWRKY63* cDNA were amplified. Subsequently, amplified fragments were ligated into the digested pCAMBIA1300 vector; the *MbWRKY63*-OE vector was constructed via homologous recombination using primers. The overexpression vector and empty vector (pCAMBIA1300) were constructed, stored at −80 °C, and a high-purity plasmid extraction kit was used to extract activated plasmids (Cat. No.: CW0500M). Artificial transformation of transgenic plants is commonly performed using *Agrobacterium tumefaciens* [92]. Plasmids were extracted from the above sequenced bacterial culture and transformed into *A. tumefaciens* using the GV3101 competent strain (Cat. No.: AC1001) from Weidi Biotechnology Co., Ltd. (Shanghai, China). The inoculum was then transferred to *Arabidopsis* plants via the floral dip method [93]. Following seed germination, T1 generation seeds transfected with the selection marker were placed on MS selection medium (50 mg/L kanamycin) for preliminary identification of transgenic *Arabidopsis*. qPCR analysis was performed on T2 generation plants to confirm the results, with WT and UL plants used as controls. Transgenic lines with high expression from the T3 generation (S1, S3, S5) were selected to breed homozygous T3-generation plants, which were used as experimental materials for further processing and analysis [94].

### 4.7. Response to Cold Stress and Assay of Relevant Physiological Metrics

Transgenic *Arabidopsis thaliana* plants (WT, UL, and T3 generations) were transferred to nutrient pots and grown in a light incubator for approximately 4 weeks, under a 16-h light, 8-h dark photoperiod. Four plants per pot were exposed to −4 °C for 8 days to simulate cold stress, followed by a 7-day recovery period under normal growth conditions [95]. For each plant line, the sample size is typically 3–5 plants. Young leaves are collected from each plant and combined to form a single biological replicate, yielding 3 biological replicates. Thus, the total sample size ranges from 9 to 15 plants, helping avoid errors caused by individual plants. Before treatment, the plants’ status (pre-treatment, post-treatment, and post-recovery) was photographed and recorded. Collect *Arabidopsis* leaf samples (WT, UL, transgenic lines S1, S3, S5); determine related physiological indexes before and after treatment, and measure chlorophyll content by extraction [96]. The activities of SOD, POD, and CAT were determined using the nitroblue tetrazolium (NBT) photoreduction method [97], guaiacol method [98], and ultraviolet (UV) absorption method, respectively. Determination of MDA content: chromatography was used, and the procedure was performed as described by Huang et al. [99]. Extraction and Determination of Pro: Pro was first extracted using the sulfosalicylic acid method, and the determination was then completed according to the experimental protocol of Li et al. [100]. The contents of H_2_O_2_ and O_2_^−^ were determined sequentially using the potassium iodide (KI) method and the hydroxylamine oxidation method, respectively [101]. Relevant parameters were measured with a conductivity meter, and the calculation was performed according to the method of Ma et al. [102].

### 4.8. Downstream Gene Expression Analysis in Transgenic Arabidopsis Under Cold Stress

The *Arabidopsis* leaves collected before and after the treatment described in Section 4.6 were used for RNA extraction. After RNA extraction, the first-strand cDNA was synthesized from the extracted RNA using WT, UL lines, and transgenic *Arabidopsis* plants (S1, S3, S5) under both −4 °C (cold stress for 8 days) and normal conditions. *MbWRKY63* is involved in the ABA signaling pathway and promotes the expression of downstream genes (*AtKIN1*, *AtCOR47*, *AtCOR15a*), thereby enhancing plant tolerance to cold environments. On the other hand, *MbWRKY63* regulates CBF cold-responsive genes through an ABA-independent signaling pathway to activate *COR47* expression; it also participates in the CBF pathway and promotes the expression of downstream *AtCBF1*, *AtCBF2*, and *AtCBF3*, ultimately enhancing plant cold hardiness. Thus, RT-qPCR detected expression of cold stress-related downstream genes (*AtKIN1*, *AtCBF1*, *AtCBF2*, *AtCBF3*, *AtCOR47*, *AtCOR15a*) in WT, UL, S1, S3, and S5. The purpose of this analysis was to investigate the expression levels of genes linked to abiotic stress responses [103]. The *Atactin* gene was selected as the reference gene. The sequences of specific primers (for target genes) and reference gene primers are provided in Table S1. The 2^−∆∆Ct^ method was applied to compute the relative expression of target genes [104].

### 4.9. Statistical Analysis

For the cold treatment in this research, three biological replicates were set per sample, with the average value applied for data analysis. The obtained index data were processed, analyzed, and plotted using GraphPad Prism software (v8.0.2.263). The experimental results were presented as mean ± standard error (SE) [105]. SPSS 26.0 was used for one-way ANOVA, with statistical differences defined (* *p* ≤ 0.05, ** *p* ≤ 0.01) [106].

## 5. Conclusions

In this study, we selected *Malus baccata* (L.) Borkh as the research material, and with the WRKY family gene *MbWRKY63* being first obtained using gene cloning technology. Phylogenetic tree analysis showed that MbWRKY63 shared the highest homology with MdWRKY63. Subcellular localization was determined using a green fluorescent protein (GFP) fusion protein expression assay, which showed that MbWRKY63 is localized to the nucleus. RT-qPCR analysis revealed that *MbWRKY63* is highly expressed in both roots and young leaves of *Malus baccata*, and its responsiveness to low-temperature environments is significantly enhanced. This expression pattern directly suggests that *MbWRKY63* may be a core response gene for *Malus baccata* to cope with low-temperature stress, thereby pointing out the research direction for subsequent functional verification. After transferring *MbWRKY63* into *Arabidopsis thaliana*, it was found that this gene effectively improves the adaptability of transgenic plants to low-temperature conditions by maintaining chlorophyll content and MDA and Pro levels, and by balancing ROS levels. This result clarifies the core role of *MbWRKY63* in regulating low-temperature tolerance and elucidates the key pathway through which it exerts its function, as revealed by physiological indicators. Downstream stress-responsive gene expression analysis revealed elevated expression of *AtKIN1*, *AtCBF1*, *AtCBF2*, *AtCBF3*, *AtCOR47*, and *AtCOR15a* in transgenic plants after cold treatment, with levels significantly higher than in WT and UL plants. It is inferred that *MbWRKY63* may enhance the freezing resistance of plants by activating the ABA-dependent signaling pathway, thereby regulating the expression of downstream low-temperature response genes, and ultimately improving the freezing resistance of plants. This mechanism analysis provides a new perspective for understanding the molecular network of WRKY genes involved in low-temperature stress regulation and analyzing the abiotic stress tolerance mechanism of *Malus baccata* and for its molecular breeding.

## Data Availability

The original contributions presented in this study are included in the article/Supplementary Material. Further inquiries can be directed to the corresponding authors.

## Supplementary Materials

The following supporting information can be downloaded at https://www.mdpi.com/article/10.3390/ijms262411997/s1.
