# Supplementary material for: Overexpression of Malus baccata WRKY63 Enhances Cold Tolerance by Increasing the Antioxidant Level Associated with ROS Scavenging
Source: Int J Mol Sci. 2025 Dec 12;26(24):11997. doi: 10.3390/ijms262411997 (PMC12732663; doi:10.3390/ijms262411997)
Supplement: Supplementary file 1 [file ijms-26-11997-s001.zip › Supplementary Figure.pdf]

```
1      ATGGGAACCAACCACAAGAGACTGATCGAGGAGCTACTAGAAGGCCGGAAGACGGCGGTGGAGCTTCAGAGTCTGCTCCACAAGCCGTT
1       M  G  T  N  H  K  R  L  I  E  E  L  L  E  G  R  K  T  A  V  E  L  Q  S  L  L  H  K  P  F
91     GGAGATCGTGAATCGGCGGAGGAGCTTTTGATGAAGATCATGACAACATTTACAGAGAGTCTCTCTGTTCTGACTGCTTCTAACGGCCAT
31      G  D  R  E  S  A  E  E  L  L  M  K  I  M  T  T  F  T  E  S  L  S  V  L  T  A  S  N  G  H
181    GAGGATCACCAGTCTGCTGCTTCCGGTGAGGTTTATCAGGTCAAGCCTGAACCTTCCCATGTGGAGCACTCGCATTGCGGTGACCGGAGC
61      E  D  H  Q  S  A  A  S  G  E  V  Y  Q  V  K  P  E  P  S  H  V  E  H  S  H  C  G  D  R  S
271    TCTGAAGGTTCCGGTGAGAGTCCAAAGACTCAGGCTTTCAAGGATCGGAGAGGTAGCTACAAGAGGAGAAAGACATCTCAATCATGGAAA
91      S  E  G  S  G  E  S  P  K  T  Q  A  F  K  D  R  R  G  S  Y  K  R  R  K  T  S  Q  S  W  K
361    GTAATCTCTACCAAAATTGAAGATGGTCAGGCGTGGAGAAAATATGGCCAAAAGATAATCCTCAAAGCTTCATATCCAAGGGCTTACTTC
121     V  I  S  T  K  I  E  D  G  Q  A  W  R  K  Y  G  Q  K  I  I  L  K  A  S  Y  P  R  A  Y  F
451    AGATGCACAAGAAAGTATGATCAAGGTTGCAAGGCAACCAAGCAGGTCCAACAAATCCAAGACAACCCACGCACGTACCAAACCACCTAC
151     R  C  T  R  K  Y  D  Q  G  C  K  A  T  K  Q  V  Q  Q  I  Q  D  N  P  R  T  Y  Q  T  T  Y
541    ATTGGCGAGCACACGTGCAGAACCATGATCAAGGCTTCTCCAATGATCATCGGCCCTGATCTTTGGCCATCTCAGACTGTTAGCTCAGAA
181     I  G  E  H  T  C  R  T  M  I  K  A  S  P  M  I  I  G  P  D  L  W  P  S  Q  T  V  S  S  E
631    TCTGGGTCCCCACATCGCCAAAACCCTAATTTCTTTGGCTCATCCTCGTCGTCCATTCCCATCGTCAAACAAGAAGATTCCAAAGTGGGG
211     S  G  S  P  H  R  Q  N  P  N  F  F  G  S  S  S  S  S  I  P  I  V  K  Q  E  D  S  K  V  G
721    ACACCAAGTGATGTAACGGACAACAATGTGTGGCTTGGTTTGAAGGATCACTTGGATTTTTCCGAGCCTACCGGAATCTGCGTGTCTTCC
241     T  P  S  D  V  T  D  N  N  V  W  L  G  L  K  D  H  L  D  F  S  E  P  T  G  I  C  V  S  S
811    AATGAGAACGTGGTTTCAAACATGCAGTGGGACGACATGGTTAAGTCTATTAATTTTGAGTGTGATATTAATTTTGATGAAGGTTTTGAT
271     N  E  N  V  V  S  N  M  Q  W  D  D  M  V  K  S  I  N  F  E  C  D  I  N  F  D  E  G  F  D
901     GCTGTTTAA
301      A  V  *
```

Figure S1. Nucleotide sequence and deduced amino acid sequence of the *MbWRKY63* gene. The start codon and stop codon are marked with a yellow underline; a WRKY conserved domain is marked with a red underline. The red boxes represent the C_2HC-type zinc finger structures of Class III A WRKY transcription factors. * denotes the termination codon.
