# Supplementary material for: Overexpression of Malus baccata WRKY63 Enhances Cold Tolerance by Increasing the Antioxidant Level Associated with ROS Scavenging
Source: Int J Mol Sci. 2025 Dec 12;26(24):11997. doi: 10.3390/ijms262411997 (PMC12732663; doi:10.3390/ijms262411997)
Supplement: Supplementary file 1 [file ijms-26-11997-s001.zip › Supplementary Table.pdf]

Supplementary Materials

Supplementary Table S1. List of primers used in this study.

Primer	Sequence (5-3')	Purpose
MbWRKY63-F	ATGGGAACCAACCACAAGAG	full-length cDNA of *MbWRKY63*
MbWRKY63-R	TTAAACAGCATCAAAACCTTCAT	full-length cDNA of *MbWRKY63*
MbWRKY63-sl F	GACGTCTTCGAGCTCGGTACC ATGGGAACCAACCACAAGAG	For subcellular localization
MbWRKY63-sl R	CATGTCGACTCTAGAGGATCC TTAAACAGCATCAAAACCTTCAT	For subcellular localization
MbWRKY63-qF	CTGACTGCTTCTAACGGCCA	qPCR
MbWRKY63-qR	GCTCCACATGGGAAGGTTCA	qPCR
MbActin-F	ACACGGGGAGGTAGTGACAA	qPCR
MbActin-R	CCTCCAATGGATCCTCGTTA	qPCR
Atactin-F	CTTGCACCAAGCAGCATGAA	qPCR
Atactin-R	CCGATCCAGACACTGTACTTCCTT	qPCR
AtKIN1(AT3G63480)-qF	AGCACAACAGGCGGGAAAG	qPCR
AtKIN1(AT3G63480)-qR	AGGCATTCTTGTTGGTCTCTGACAT	qPCR
AtCBF1(AT4G25490)-qF	GAGACGATGGTGGAAGCTATTT	qPCR
AtCBF1(AT4G25490)-qR	AGCATGCCTTCAGCCATATTA	qPCR
AtCBF2(AT4G25470)-qF	GACCTTGGTGGAGGCTATTT	qPCR
AtCBF2(AT4G25470)-qR	ATCCCTTCGGCCATGTTATC	qPCR
AtCBF3(AT4G25480)-qF	GACGTTGGTGGAGGCTATTT	qPCR
AtCBF3(AT4G25480)-qR	AGCATCCCTTCTGCCATATTAG	qPCR
AtCOR47(AT1G20440)-qF	CGTTGTTCTTGTACTCCTCAGCCAT	qPCR
AtCOR47(AT1G20440)-qR	GCTCTCCATGAGCTGGTGAT	qPCR
AtCOR15a(AT2G42540)-qF	AGACAAATACGCCGAAGA	qPCR
AtCOR15a(AT2G42540)-qR	CATACAGGACCCTATCACG	qPCR
